# Exploring the genome of *Lactobacillaceae* spp. Sy-1 isolated from *Heterotrigona itama* honey

**DOI:** 10.7717/peerj.13053

**Published:** 2022-03-23

**Authors:** Syariffah Nuratiqah Syed Yaacob, Fahrul Huyop, Mailin Misson, Roswanira Abdul Wahab, Nurul Huda

**Affiliations:** 1 Department of Bioscience, Faculty of Science, Universiti Teknologi Malaysia, Skudai, Johor, Malaysia; 2 Enzyme Technology and Green Synthesis Group, Universiti Teknologi Malaysia, Skudai, Johor, Malaysia; 3 Biotechnology Research Institute, Universiti Malaysia Sabah, Jalan UMS, Kota Kinabalu, Malaysia; 4 Department of Chemistry, Faculty of Science, Universiti Teknologi Malaysia, Skudai, Johor, Malaysia; 5 Faculty of Food Science and Nutrition, Universiti Malaysia Sabah, Jalan UMS, Kota Kinabalu, Malaysia

**Keywords:** Stingless bee *Heterotrigona itama*, Folic acid, Honey, Functional genome prediction, *Lactobacillaceae*, Fructophilic lactic acid bacteria

## Abstract

**Background:**

Honey produced by *Heterotrigona itama* is highly preferred among consumers due to its high-value as a functional food and beneficial lactic acid bacteria (LAB) reservoir. Fructophilic lactic acid bacteria (FLAB) are a group of LAB with unique growth characteristics and are regarded as promising producers of bioactive compounds. Hence, it is not surprising that LAB, especially FLAB, may be involved with the excellent bioactivity of *H. itama* honey. With the trending consumer preference for *H. itama* honey coupled with increasing awareness for healthy food, the genomic background of FLAB isolated from this honey must, therefore, be clearly understood. In this study, one FLAB strain designated as Sy-1 was isolated from freshly collected *H. itama* honey. Its FLAB behavior and genomic features were investigated to uncover functional genes that could add value to functional food.

**Methods:**

The fructophilic characteristics of strain Sy-1 were determined, and the genome was sequenced using Illumina iSeq100 and Oxford Nanopore. The average nucleotide identity and phylogenetic analyses based on 16S rRNA, 92 core genes, and whole-genome sequence were performed to unravel the phylogenetic position of strain Sy-1. NCBI Prokaryotic Genome Annotation Pipeline annotated the genome, while the EggNOG-mapper, BLASTKoala, and GHOSTKoala were used to add functional genes and pathways information.

**Results:**

Strain Sy-1 prefers D-fructose over D-glucose and actively metabolizes D-glucose in the presence of electron acceptors. Genomic annotation of strain Sy-1 revealed few genes involved in carbohydrate transport and metabolism, and partial deletion of *adhE* gene, in line with the characteristic of FLAB. The 16S rRNA gene sequence of strain Sy-1 showed the highest similarity to unknown LAB species isolated from the gut of honeybees. The phylogenetic analyses discovered that strain Sy-1 belonged to the *Lactobacillaceae* family and formed a separate branch closer to type strain from the genera of *Acetilactobacillus* and* Apilactobacillus.* The ANI analysis showed the similarity of the closest relative, *Apilactobacillus micheneri* Hlig3^T^. The assembled genome of Sy-1 contains 3 contigs with 2.03 Mbp and a 41% GC content. A total of 1,785 genes were identified, including 1,685 protein-coding genes, 68 tRNA, and 15 rRNA. Interestingly, strain Sy-1 encoded complete genes for the biosynthesis of folate and riboflavin. High-performance liquid chromatography analysis further confirmed the high production of folic acid (1.346 mg/L) by Sy-1.

**Discussion:**

Based on phylogenetic and biochemical characteristics, strain Sy-1 should be classified as a novel genus in the family of *Lactobacillaceae* and a new member of FLAB. The genome information coupled with experimental studies supported the ability of strain Sy-1 to produce high folic acid. Our collective findings support the suitable application of FLAB strain Sy-1 in the functional food and pharmaceutical industries.

## Introduction

Lactic acid bacteria (LAB), mainly from the family of *Lactobacillaceae,* have been consumed by humans since ancient times for various health benefits. Certain LAB displays high biosynthetic ability and can utilize raw materials to produce exopolysaccharides, bioactive peptides, and vitamins such as folic acid and riboflavin. Such vitamins are essential for humans to maintain normal metabolic activities and optimal health (Capone & Sentongo, 2019). Unlike humans and animals that cannot synthesize most vitamins, bacteria can naturally produce such metabolites (Wang et al., 2021a; Wang et al., 2021b). The inherent ability of bacteria to produce these metabolites might be helpful to consumers who are becoming more health conscious and making better food choices (Verain et al., 2012). These bacteria can produce fermented bio-enriched food by synthesizing B-group vitamins, notably riboflavin and folic acids (Levit, De Giori GS & LeBlanc, 2019; Molina et al., 2012). Vitamin-producing LAB is distinctively better over chemically synthesized ones and enhances the nutritional content of food (Capozzi et al., 2012; LeBlanc et al., 2011). Due to environmental and cost concerns, chemical vitamin production is being phased out in favor of fermentation techniques (Yuan et al., 2019), which application has found its way in the dairy industry where vitamin-producing strains are added to fermented products (Li et al., 2017; Arena et al., 2014).

In April 2020, the taxonomy of the genus *Lactobacillus* was revised to reclassify species formerly described as *Lactobacillus* species into 25 genera that comprise phylogenetically related bacteria that share major metabolic traits and ecological origin (Zheng et al., 2020). Since 18 January 2022, the EzBioCloud database showed that the genera of the *Lactobacillaceae* family had been updated to 31 (Zheng et al., 2020). The fructophilic LAB (FLAB) is a newly explored potential LAB with unique functional traits, showing a preference for D-fructose over D-glucose as a carbon source (Endo, 2012; Filannino et al., 2016). FLAB metabolically belongs to a group of heterofermentative LAB, but the final products from D-glucose metabolism clearly distinguish FLAB from other heterofermentative LAB. While lactate, ethanol, and CO_2_ are the major final products for heterofermentative LAB, in the case of FLAB, ethanol is substituted with acetate. These phenotypic characteristics place FLAB in a separate class within the LAB group that generally prefers D-glucose and anaerobic conditions for growth. In fact, the ability of FLAB to grow so well under the above-said condition because they have a complete or partial deletion of the *adhE* gene that encodes for a bifunctional alcohol/acetaldehyde dehydrogenase, AdhE. Contrasting to the heterofermentative LAB, which relies on the AdhE enzyme to maintain the NAD/NADH balance during D-glucose metabolism, the FLAB uses external electron acceptors to oxidize NADH to NAD^+^. FLAB is also a poor carbohydrate fermenter and metabolizes only a few carbohydrates (Maeno et al., 2019). This bacterial group is ubiquitous in fructose-rich environments, such as flowers, fruits, gastrointestinal tracts of various insects, including the honeybee, honeybee larvae, bee pollen, and fresh honey (Endo, 2012). To date, the FLAB members are represented by all species of the genus *Fructobacillus* and several species from the *Apilactobacillus* genus (*Apilactobacillus apinorum*, *Apilactobacillus kunkeei Apilactobacillus micheneri, Apilactobacillus quenuiae, Apilactobacillus timberlakei*), *Fructilactobacillus florum* and *Levilactobacillus brevis* (Endo et al., 2010; Maeno et al., 2021).

Malaysia is home to 50 distinct species of stingless bees belonging to seven genera (Engel & Rasmussen, 2017), with the *Heterotrigona itama* (*H. itama*) being the most popular. Honey produced by *H. itama* bees is well-documented to show good biological properties, *viz.* antioxidant, anti-inflammatory, anti-bacterial, and anti-ulcer properties (Kek et al., 2017; Majid, Ellulu & Abu Bakar, 2020). Thus, it is not surprising that LAB, especially FLAB harbouring *H. itama* honey may be involved in its excellent bioactivity. With increasing consumer preference for *H. itama* honey coupled with increasing awareness for healthy food, the genomic background of FLAB isolated from this honey must, therefore, be clearly understood.

In this study, a single FLAB was isolated from freshly collected *H. itama* honey and designated as strain Sy-1. The bacterial isolate was characterized *via* genotypic and phenotypic approach, and its taxonomic position in the family of *Lactobacillaceae* was identified*.* Gene components in the genome of strain Sy-1 were compared with the phylogenetically closest LAB species to gain an insight into its unique genes. Based on the collective findings, strain Sy-1 is proposed to represent a novel genus in the family *Lactobacillaceae* and a new member of FLAB.

## Materials & Methods

### LAB isolation and DNA extraction

Fresh honey was collected from the enclosed pot of *H. itama* bees at Kulai, Johor, Malaysia (103°38′21.822″N, 1°39′25.1676″E) and was placed into a sterilized bottle and proceeded with the isolation process on the same day. A total of 1 g of *H. itama* honey was suspended in 10 mL of MRS broth (Merck, Darmstadt, Germany), and the mixture was vortexed for 10 s. A ten-fold serial dilution was performed, and 100 µL from each dilution was spread onto MRS agar supplemented with 20 g/L fructose and 0.8% CaCO_3_. The plates were incubated at 30 °C for 3 to 4 days under aerobic conditions. Individual colonies were selected based on clear zone formed from hydrolysis of the CaCO_3_ by lactic acid and were sub-cultured to obtain pure isolates. Only gram-positive isolates that exhibited negative-catalase activity were kept in MRS broth supplemented with 20% (v/v) glycerol and stored at −80 °C. A scanning electron microscope (Hitachi, JEOL, Japan) was used to characterize the cellular morphology of isolated FLAB. ZymoBIOMICS™ DNA Miniprep Kit was used to extract the genomic DNA. The 16S rRNA gene was amplified as previously described in Syed Yaacob et al. (2018). The amplified 16S rRNA gene was purified by QIAquick PCR purification kit (Qiagen, Hilden, Germany) and was sequenced by Apical Scientific (Selangor, Malaysia).

### Sequencing strategy and assembly

The whole-genome of strain Sy-1 was sequenced by a hybrid strategy of Illumina iSeq100 and MinION Nanopore Sequencing. A 300 ng of genomic DNA of Sy-1 was processed using the NEBNext^®^ Ultra™ II FS DNA Library Prep Kit for Illumina. The constructed library was subsequently quantified using the Denovix high-sensitivity fluorescence quantification kit followed by sequencing on iSeq100 (Illumina, California, USA) using the run configuration of 1 × 300 bp. Raw reads from Illumina iSeq100 were adapter-trimmed using Trimmomatics v0.39 (Bolger, Lohse & Usadel, 2014). Approximately 1 µg of genomic DNA was processed using the Nanopore LSK109 kit for nanopore sequencing. The constructed library was loaded onto a SpotON Flow Cell Rev D and sequenced for 3 h. Base-calling of the raw fast5 file used the high accuracy mode Guppy v4, and the Flye v2.7.1 performed de-novo assembly of the nanopore reads. The Illumina reads were subsequently aligned to the raw Flye-assembled contigs using bwa mem (bwa−0.7.17-r1188) (Li & Durbin, 2009). A total of 42x Illumina genome coverage (788,813 trimmed single-end reads, 8,3480,593 bp) and 170x Nanopore genome coverage (56,657 reads, 344,455,836 bp) were used for hybrid genome assembly. Finally, a single round of genome polishing *via* Pilon v1.23 (Walker et al., 2014) was performed using the sorted BAM alignment.

### Phylogenetic analysis and average nucleotide identity (ANI)

The resultant 16S rRNA sequence was aligned with the NCBI non-redundant database using the BLASTn program and confirmed by accessing the EZbiocloud server to determine the phylogenetic position of strain Sy-1. Phylogenetic analysis was confirmed using the 16S rRNA gene sequences of type strains scattered in the 31 genera of the *Lactobacillaceae* family. The phylogenetic trees were constructed using MEGA11 (Tamura, Stecher & Kumar, 2021) using the maximum-likelihood (ML) method with bootstrap analysis based on 1,000 replications. Additionally, multigene analysis of the phylogenetic relationship among strain Sy-1 and other members of *Lactobacillaceae* was performed using the UBCG pipeline (Kim et al., 2021). For building the phylogenomic tree, a total of 65 type strains scattered in the 31 genera of the *Lactobacillaceae* family were selected as an input which was retrieved with the help of PRODIGAL v2.6. HMMER3 v3.2.1 was used to scan through the *Lactobacillaceae* HMMs in each respective genome set which were eventually aligned and concatenated with TRIMAL v1.4 and MUSCLE v3.8. The GToTree-generated fasta file of an aligned single-copy gene (SCG) was used as input to create a phylogenomic tree on MEGA11 software (Tamura, Stecher & Kumar, 2021), and evolutionary distances were calculated using the Maximum Likelihood method and JTT matrix-based model. The average nucleotide identity (ANI) between Sy-1 and type strains from thirty-one genus members of *Lactobacillaceae* were determined using the OrthoANI algorithm from the JSpeciesWS tool.

### Genome annotation

The complete genome of strain Sy-1 was annotated by NCBI Prokaryotic Genome Annotation Pipeline (PGAP) (Tatusova et al., 2016). GC content was determined by whole-genome sequencing analysis. Protein coding genes were predicted by GeneMarkS^+^, while tRNA and rRNA by tRNA-scan-SE and RNAmmer (Lowe & Chan, 2016). The orthologous annotations were computed using EggNOG-mapper v2 (Cantalapiedra et al., 2021). BlastKOALA and GhostKOALA were used to reconstruct the metabolic pathways (Kanehisa, Sato & Morishima, 2016). Clustered Regularly Interspaced Short Palindromic Repeat (CRISPR) were identified using CRISP-cas ++ 1.1.2 (Grissa, Vergnaud & Pourcel, 2007), while PHASTER (Arndt et al., 2016) and IslandViewer v4 (Bertelli et al., 2017) identified the prophages and genomic islands, respectively. The presence of plasmids was investigated using PlasmidFinder v2.1 (Carattoli & Hasman, 2020). The 16S rRNA gene and the whole-genome sequence of strain Sy-1 are publicly accessible in the NCBI database *via* GenBank accession numbers MW715007 and JACCIU000000000, respectively. The culture of strain Sy-1 was deposited in the Microbial Culture Collection (UNICC), Institute of Bioscience, Universiti Putra Malaysia, and the accorded accession number is UPMC 1412.

### Carbohydrate metabolism and analysis of CAZymes

The carbohydrate metabolism of strain Sy-1 was analyzed using API^^®^^ 50 CHL kit (BioMérieux). Growth behavior on D-glucose and D-fructose as well as the requirement of external electron acceptors for D-glucose assimilation were determined in glucose-yeast broth (GYP), fructose-yeast broth (FYP), and GYP broth supplemented with 10 g/L of pyruvate (GYP-P) and GYP broth supplemented with 10 g/L fructose (GFYP) under static condition. Oxygen utilization as an electron acceptor was determined in GYP broth under an aerobic environment with incubation on an orbital shaker (120 rpm) at 30 °C for four days. Growth was monitored at 620 nm with a visible spectrophotometer (Nanocolor^^®^^ vis, Macherey-Nagel, German). Anaerobic growth on D-glucose was determined by streaking the isolate onto GYP agar and then incubated in an anaerobic system (BD GasPak™ 150 system). Ethanol production was quantified using gas chromatography equipped with a flame ionization detector (GC-FID) after five days of incubation in GYP broth at 30 °C. Mannitol production from D-fructose was determined in GFYP broth using ultra-high-performance liquid chromatography coupled with an evaporative light scattering detector (UHPLC-ELSD) system (Shimadzu, Kyoto, Japan), as described by Wang et al. (2021a) and Wang et al. (2021b). The putative genes for carbohydrate-active enzymes (CAZymes) encoded in the genome of strain Sy-1 were annotated (blastn) using dbCAN2 meta server (Zhang et al., 2018), including HMMER (dbCAN CAZyme domain HMM database with *E*-value < 1e−15), DIAMOND (CAZy database with *E*-value < 1e−102) and Hotpep (short conserved motifs in the PRR library database with frequency > 2.6, Hits > 6).

### Comparative genomic analyses

To define the pan genome, all genes were clustered into orthogroups using OrthoFinder (version 4.11.0) (Emms & Kelly, 2019). Here, a core orthogroup is defined as an orthogroup present in more than 95% of a set genome. All other orthogroups are defined as accessory orthogroups. An upset plot was created using the R package UpsetR (Conway, Lex & Gehlenborg, 2017). Unique orthogroups belonging to strain Sy-1 were further annotated using EggNOG-mapper v2 (Cantalapiedra et al., 2021) and visualized using Origin software.

### Detection of folic acid and riboflavin from strain Sy-1

Folic acid and riboflavin production by the strain Sy-1 were quantified using high-performance liquid chromatography (HPLC) Infinity 1290 (Agilent Technologies, USA) equipped with a diode array detector (DAD). Chromatographic separation was performed on the Zorbax C18 column with the temperature maintained at 30 °C. The mobile phase and the isocratic elutions were performed following the method described by Rakuša, Grobin & Roškar (2021). For sample preparation, strain Sy-1 was grown overnight at 30 °C in MRS broth supplemented with 20 g/L of fructose. The following day, the bacteria culture was centrifuged (4,000 rpm, 4 °C, 20 min) to separate the produced vitamins from the bacterial pellet. The supernatant was filter-sterilized through a 0.22 µm filter and injected into the HPLC system. Folic acid and riboflavin contents produced by strain Sy-1 were identified by comparing with the retention time of standards. Standard solutions of folic acid and riboflavin were prepared at various concentrations for quantitative analysis. The calibration curves are then built utilizing the corresponding peak areas of the analytes. The measurements were triplicated, and the results were presented as mean ± standard deviation.

### Antibiotic-resistant assessment

The MRS agar was individually spread with 0.1 ml (10^6^ CFU/ml) of 16 h old Sy-1 culture. Antibiotic discs sulphafurazole (300 µg), ciprofloxacin (5 µg), tetracycline (30 µg), chloramphenicol (30 µg), and gentamicin (10 µg) were placed on MRS agar surface and incubated for 24 h at 30 °C. The resistance and sensitivity of the strain were determined according to the clear zone formation. Detection of antibiotic resistance genes was made using the Resfam, ResFinder (Bortolaia et al., 2020), Antibiotic Resistance Genes Database (ARDB), and Comprehensive Antibiotic Resistance Database (CARD) (Alcock et al., 2020).

### Results and Discussion

#### Identification and fructophilic characteristic of Sy-1

**Figure 1:**
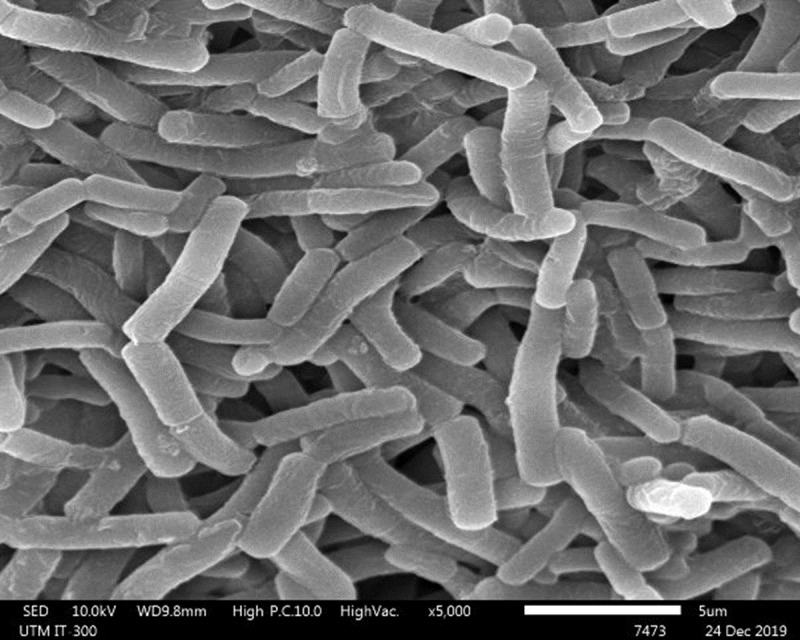
The scanning electron microscopy of strain Sy-1. The scale bar represents 5 µm.

**Figure 2:**
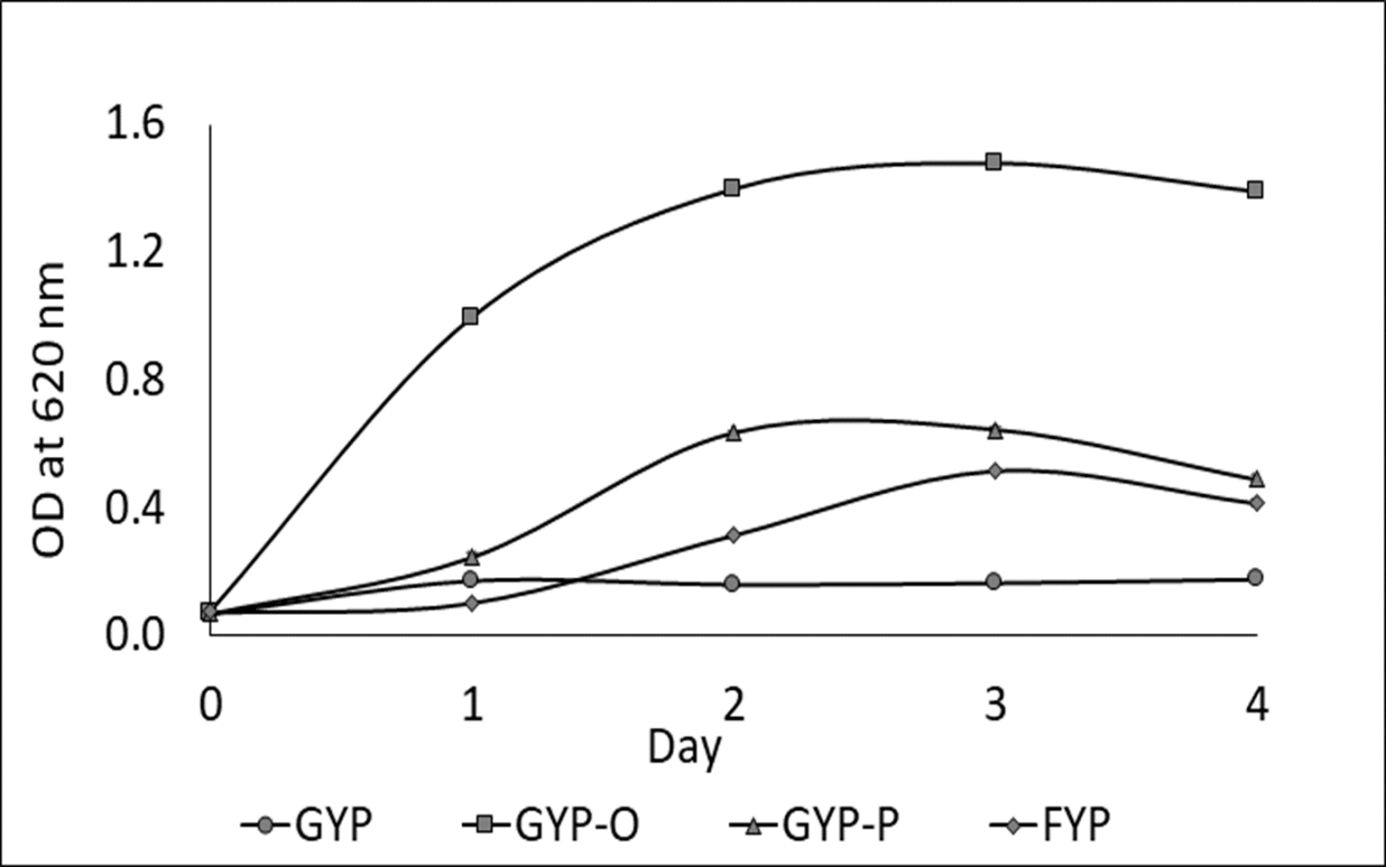
Growth behavior of strain Sy-1 in GYP broth (O), FYP broth (♢), GYP-P broth (Δ), and GYP broth under aerobic conditions (□). Data indicate mean ± standard deviation (error bars).

One LAB strain designated as Sy-1 was isolated from *H. itama* honey (5 CFU/g). Gram-staining and SEM images (Fig. 1) showed that strain Sy-1 is a gram-positive with rod-like structure and has a mean length of 6.18 µm ± 0.92. The Sy-1 bacterium is non-motile, non-spore-forming, and exhibits catalase-negative activities. Based on the API50CHL analysis, strain Sy-1 could metabolize a limited number of carbohydrates, namely D-fructose, D-sucrose, D-glucose, D-maltose, D-raffinose, D-turanose, and methyl-D-glucopyranoside. The finding was expected as fructose, sucrose, glucose, and maltose are the four predominant sugars present in *H. itama* honey. Strain Sy-1 grew well in D-fructose-yeast extract-peptone (FYP) broth but poorly in D-glucose-yeast extract-peptone (GYP) broth (Fig. 2). Notably, the addition of pyruvate (GYP-P) and aerobic culturing (GYP-O_2_) markedly enhanced growth on D-glucose. The outcome suggests that the bacteria require external electron acceptor(s) to metabolize D-glucose. Mannitol was produced at a level of 5.471 mM by the strain Sy-1 during growth in GYP broth supplemented with D-fructose after an overnight static incubation. A low quantity of ethanol (0.0236 mM) from the glucose fermentation was detected, thereby consistent with the low ethanol production by FLAB strains (0.050–0.640 mM) (Filannino et al., 2016; Maeno et al., 2016; Maeno et al., 2019).

**Figure 3:**
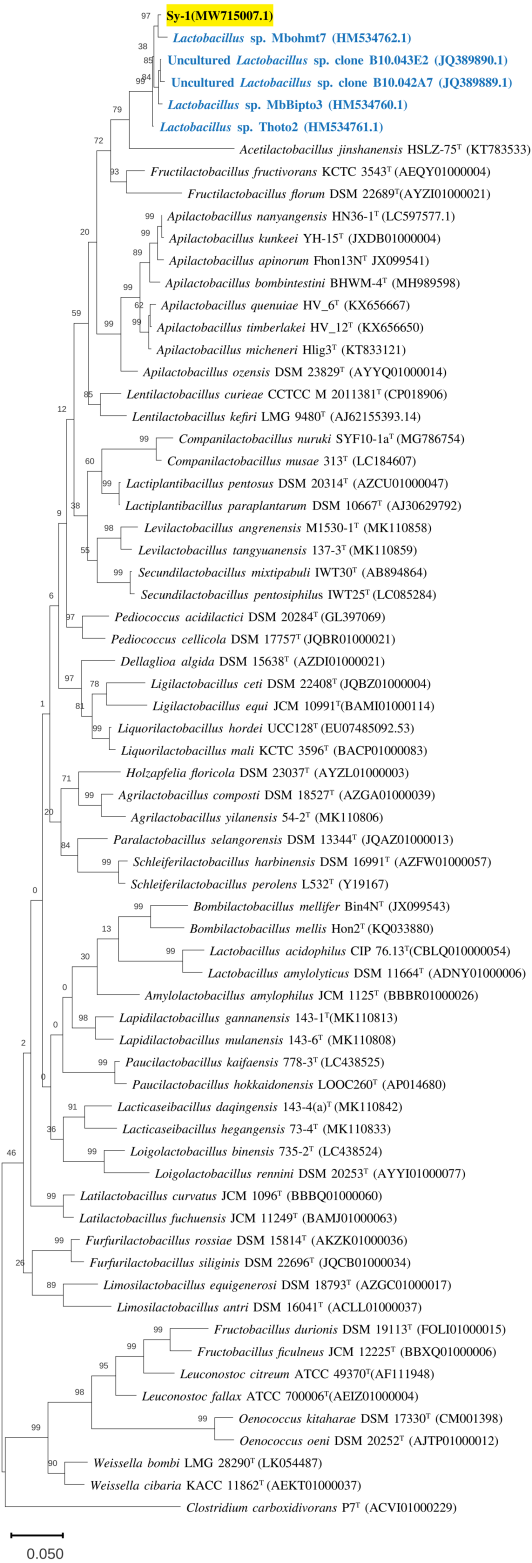
The phylogenetic tree based on 16S rRNA. The tree was inferred by using the Neighbor-Joining method in MEGA11. The percentages of replicate tree in which the associated taxa clustered together in bootstrap test (1,000 replicates) are shown above the branches. The percentages of replicate tree in which the associated taxa clustered together in bootstrap test (1,000 replicates) are shown above the branches. The tree is drawn to scale, 0.05 with branch lengths in the same units as those of the evolutionary distances used to infer the phylogenetic tree. The evolutionary distances were computed using the Jukes-Cantor method and are in the units of the number of base substitution per site. This analysis involved 68 nucleotide sequences and there were a total of 1,588 positions in the final dataset.

Bifunctional ADH/ALDH proteins are vital enzymes encoded by the *adhE* gene for D-glucose assimilation and the resultant ethanol synthesis in the phosphoketolase pathway of heterofermentative LAB (Maeno et al., 2016; Maeno et al., 2019). In LAB, the *adhE* gene is translated into a protein containing 850–900 amino acids containing C-terminal ADH and N-terminal ALDH domains (Maeno et al., 2016). Strain Sy-1 encodes incomplete *adhE* gene encoding 458 amino acids that contain only ALDH domain **(S1)** but not the ADH domain. The lack of ADH domain and the resultant loss of ADH/ALDH activity with low ethanol production causes insufficient NAD^+^ regeneration. This is because the ethanol production from acetyl-CoA uses the ALDH domain, while ADH is the key system for oxidizing NADH to NAD^+^. Interestingly, of the four fructophilic *Apilactobacillus* species, three species *A. kunkeei*, *A. timberlakei*, and *A. micheneri*, possess partial *adhE*, conserving only the ALDH domain, while *A. apinorum* completely lacks the gene. Conversely, the *A. ozensis* possesses the intact gene (Maeno et al., 2021). Sy-1 strain grew well in the presence of pyruvate under aerobic conditions; hence this verified the roles of oxygen and pyruvate as external electron acceptors to oxidize NADH to NAD^+^ and keep them in balance. Thus, the above phenotypic and metabolic behaviors collectively confirmed that Sy-1 being an obligate FLAB.

#### Taxonomic affiliation of strain Sy-1

**Figure 4:**
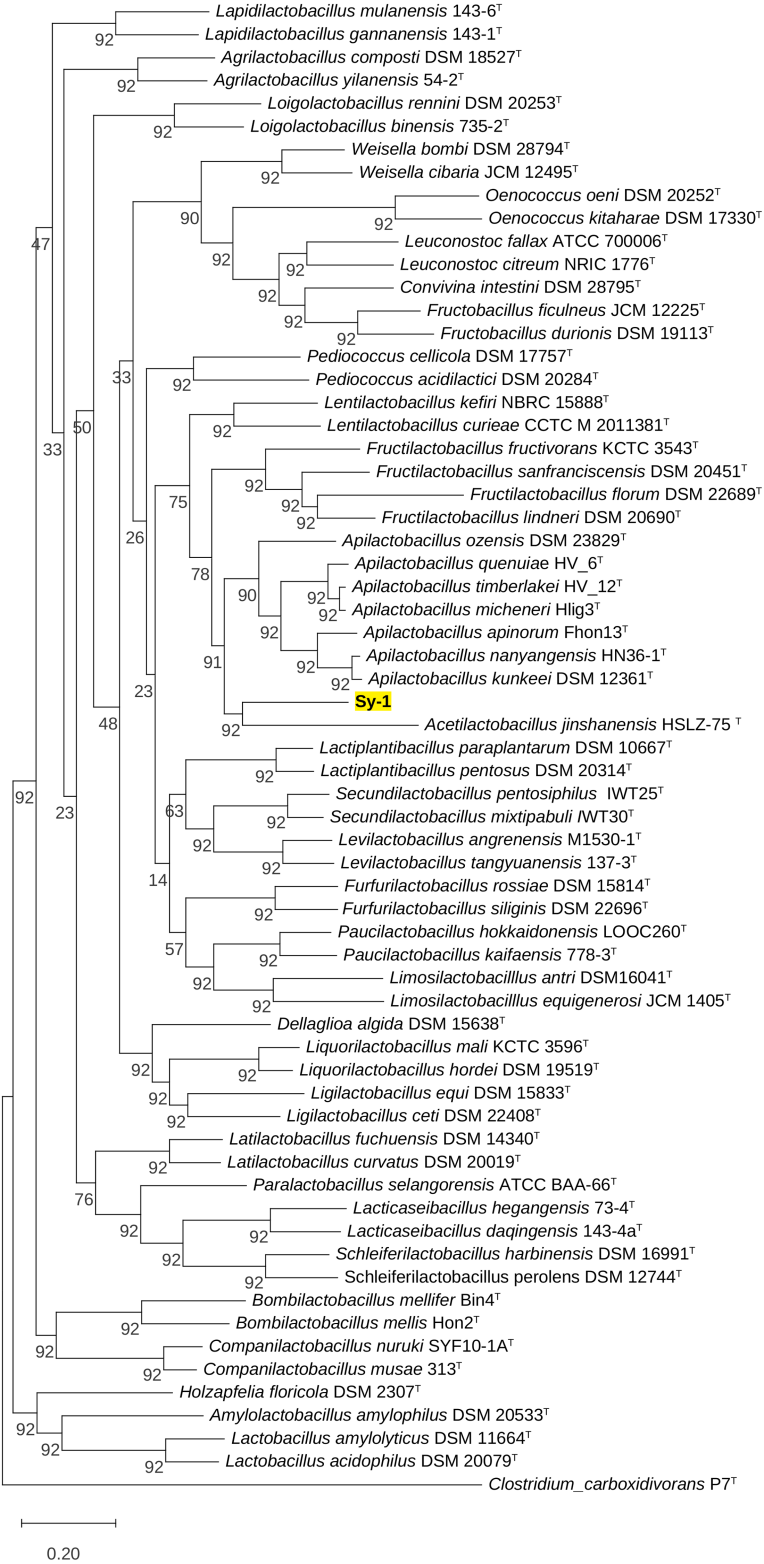
Maximum likelihood phylogenetic tree reconstructed using the 92 bacterial core genes indicating the relationship of strain Sy-1 with thirty-one genera within the family of *Lactobacillaceae*. *Clostridium carboxidivorans* P7^T^ within the family *Clostridiaceae* was used as an outgroup. Bootstrapping was carried out for 1,000 replicates. Each node indicated bootstrap value greater than 50%. Bar, 2% sequence divergence.

**Figure 5:**
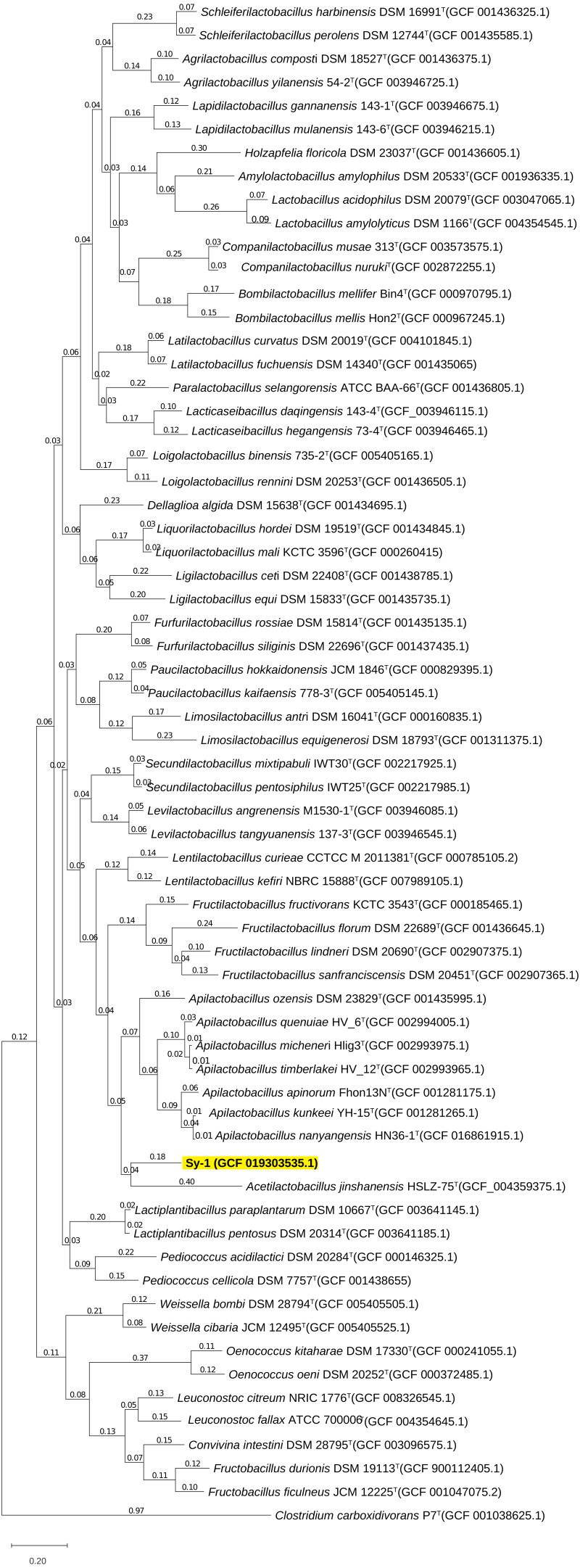
The phylogenetic tree based on whole genome sequence indicating the relationship of strain Sy-1 with the type strains of thirty-one genera within the family of *Lactobacillaceae*. The tree was inferred using the Maximum likelihood method and JTT matrix-based model. The tree is drawn to scale, with branch lengths measured in the number of substitutions per site (above branches). This analysis involved 64 members of *Lactobacilloceae* with a total of 22,730 positions in the final dataset.

BLAST search against sequences in the NCBI database revealed that strain Sy-1 showed the closest 16S rRNA sequence similarity to *Lactobacillus* sp. strains Thoto2 (99.03%), Mbohmt7 (98.76%), and MbBipto3 (98.48%) isolated from the gut of sting bee *Apis* species. These were followed by uncultured *Lactobacillus* sp. clones B10.043E2 (98.60%) and B10.043E2 (98.33%) isolated from the gut of stingless bees *Melipona panamica.* According to the EzBiocloud database, type strain *Lentilactobacillus curieae* CCTCC M 2011381^T^ isolated from tofu brine gave the highest 16S rRNA sequence similarity (93.57%) to strain Sy-1. However, the value (93.57%) was significantly lower than the 98.5–98.7% cut-off value used to delineate a bacterial species (Kim et al., 2014). A phylogenetic tree based on the 16S rRNA gene sequences (Fig. 3) showed that strain Sy-1 belonged to the family *Lactobacillaceae* and is clustered with the above-mentioned *Lactobacillus* sp. Thoto2, Mbohmt7, MbBipto3, and uncultured *Lactobacillus* sp. clone B10.043E2 and B10.043E2. Additionally, phylogenetic trees based on 92 bacterial core genes (Fig. 4) and whole-genome sequences (Fig. 5) established that strain Sy-1 formed an independent branch, separated from type strains affiliated with *Acetilactobacillus* and *Apilactobacillus* genera. The above-mentioned phylogenetic data imply that strain Sy-1 could be assigned to a novel genus scattered in the *Lactobacillaceae* family. From the ANI analysis **(S2)**, *Apilactobacillus micheneri* Hlig3^T^ isolated from the bee gut gave the highest ANI hits corresponding to 69.33%. However, the value was substantially lower than the 75% threshold value for genus circumscription (Barco et al., 2020). Taken together, the combination of phylogenetic findings and ANI results supported our findings that strain Sy-1 belonged to a novel genus within the family of *Lactobacillaceae.*

#### General genomic features of Sy-1

Whole-genome sequencing of strain Sy-1 resulted in 3 contigs that gave a total of 2,035,175 bases. The genome size for Sy-1 is the largest compared to genera *Acetilactobacillus* (1.61 Mbp) and *Apilactobacillus* (∼1.51 Mbp). Strain Sy-1 showed a GC content of 41%, the highest value instead of 39.7% in *Acetilactobacillus* and ∼33.1% in *Apilactobacillus* spp. The PGAP annotator predicted 1785 genes in the genome of Sy-1, with 273 genes (16.11%) representing hypothetical proteins. In addition, 1036 genes were assigned to different pathways according to the KEGG database. A total of 15 rRNA operons comprised of 16S (5), 23S (5), and 5S (5) genes were identified and oriented in the same direction as DNA replication. Additionally, 15 tandem repeats were identified in the genome. The study identified 68 tRNA encoding sequences that represented 20 amino acids, all of which were scattered around the genome, with one specific cluster of 18 tRNA genes at ∼1,621 kb. The main genome features of strain Sy-1 are summarized in Table 1.

**Table 1:** Key features of the strain Sy-1 genome.

**Features**	**Values**
Family	*Lactobacillaceae*
Strain	Sy-1
Isolation sources	Malaysian *Heterotrigona itama* honey
Genome size (bp)	2,035,175
Number of contigs	3
GC content (%)	41
Total genes predicted	1,785
Protein coding genes	1,695
CDSs	1,700
With COGs	1,717
Connected to KEGG pathway	1,036
Plasmid	Not detected
rRNAs	15
tRNAs	68
Tandem repeat	15
Total ORFs	1,911
Total predicted CDSs	1,734
Total hypothetical proteins	573
CRISPRs	2 (cas-1, cas-9)
Transposases	1

#### Functional categories of Sy-1 and FLAB members

**Figure 6:**
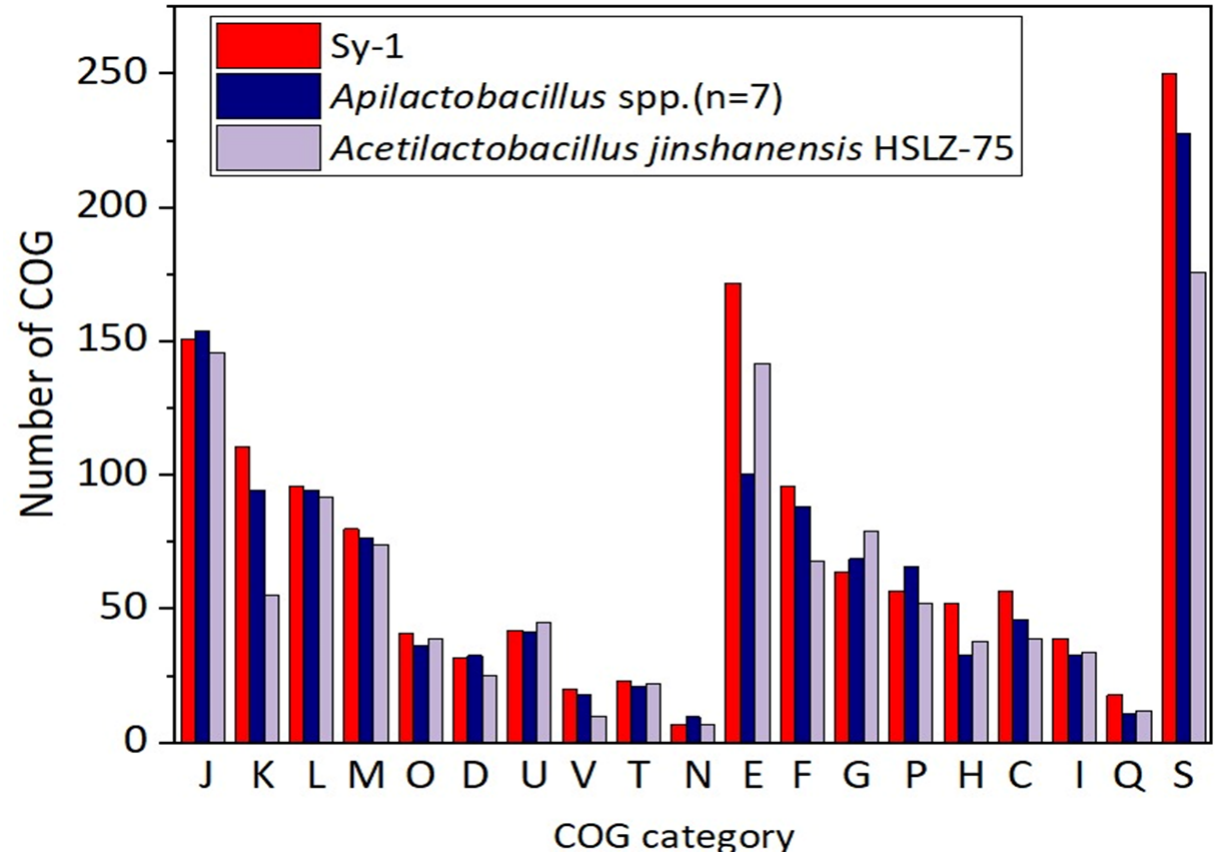
Comparison of the distribution of genes by COG functional category for strain Sy-1, *Acetilactobacillus jinshanensis* HZL-75_T_ and *Apilactobacillus* spp. COG functional categories: J: translation, ribosomal structure, and biogenesis; K: transcription; L: DNA replication, recombination, and repair; M: cell envelope biogenesis, outer membrane; O: post-translational modification, protein turnover, and chaperones; D: cell division and chromosome partitioning; U: intracellular trafficking and secretion; V: defense mechanisms; T: signal transduction mechanisms; N: cell motility and secretion; E: amino acid transport and metabolism; P: inorganic ion transport and metabolism; H: coenzyme metabolism; C: energy production and conversion; G: carbohydrate transport and metabolism; F: nucleotide transport and metabolism; I: lipid metabolism; Q: secondary metabolites biosynthesis, transport, and catabolism; S: function unknown.

Figure 6 depicts the distributions of proteins in the clusters of orthologous group (COG) functional categories for Sy-1, *Acetilactobacillus jinshanensis* HSLZ-75^T^, and *Apilactobacillus* spp. Of the 1,700 coding sequences (CDS) belongs to strain Sy-1, a total of 1,412 CDS were explicitly assigned to clusters of COG families comprising 19 functional categories, placing the Sy-1 genes in the “poorly characterized” category (S-function unknown, 250 genes, 17.7% of all genes). The fact that these genes have no annotation confirms this strain’s novelty and unexplored potential. The authors noticed that number of genes categorized in amino acid transport and metabolism (E), transcription (K), coenzyme transport and metabolism (H), and energy production and conversion (C) for strain Sy-1 were higher compared to *Acetilactobacillus jinshanensis* and species from *Apilactobacillus*. These were the probable reasons that strain Sy-1 was placed separately from *Apilactobacillus* and *Acetilactobacillus* branches. In addition, strain Sy-1 and *Apilactobacillus* spp. harboring lesser genes responsible for carbohydrate transport and metabolism (G) than *Acetilactobacillus jinshanensis*.

#### Genome comparison

**Figure 7:**
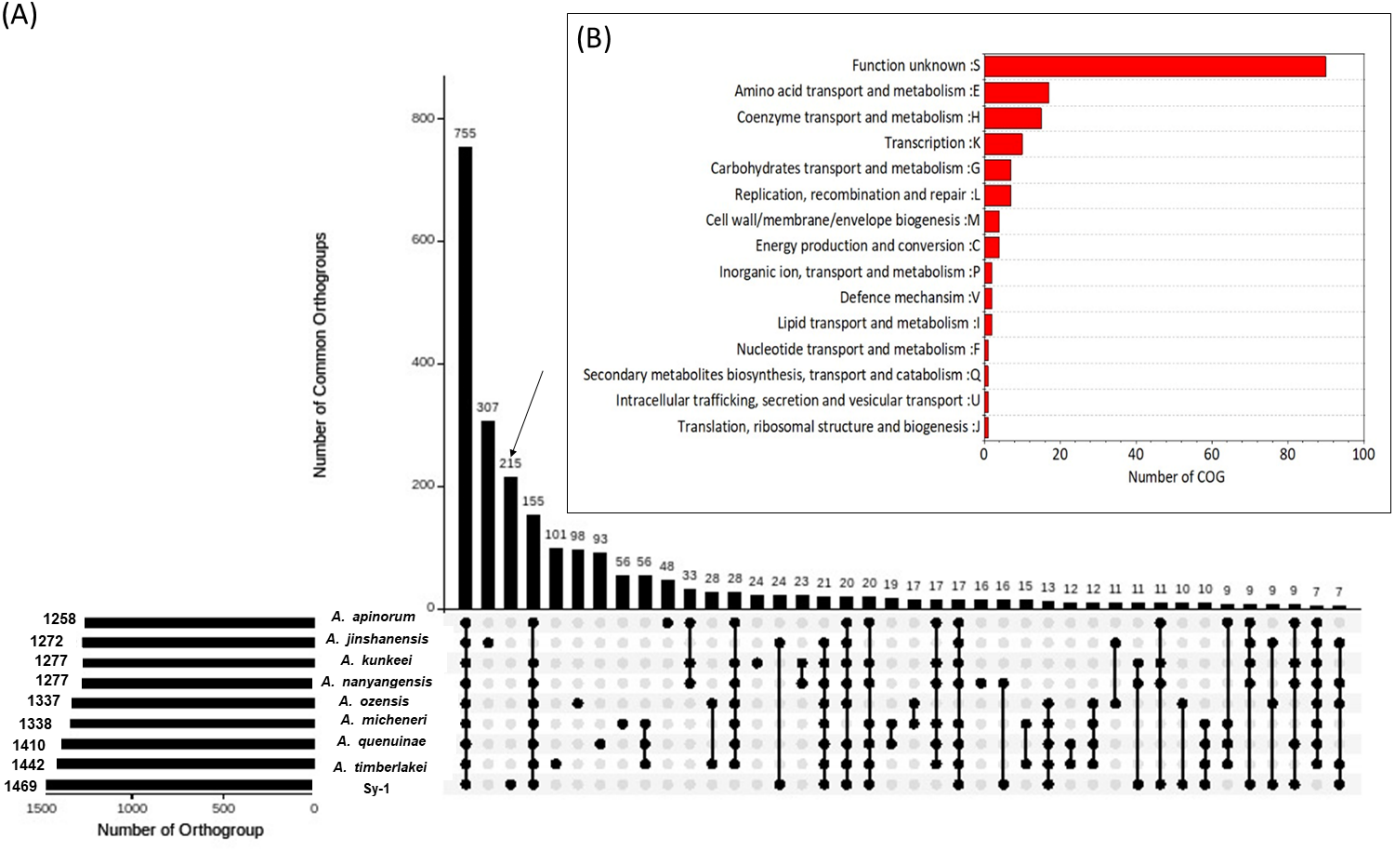
(A) Upset plot comparing shared orthogroup of Sy-1, *Apilactobacillus* spp (*n* = 7) and *Acetilactobacillus jinshanensis* HSZL-75_T_ (B) Cluster of Orthologous (COG) of the unique orthogroup of strain Sy-1.

Orthology relationships between proteins belong to strain Sy-1, *Acetilactobacillus jinshanensis* HSLZ-75^T^ and seven species (type strain)-derived *Apilactobacillus* genus (*A. ozensis* DSM 23829^T^, *A. quenuiae* HV-6^T^, *A. micheneri* Hlig3^T^, *A. timberlakei* HV-12^T^, *A. apinorum* Fhon13N^T^, *A. kunkeei* YH-15^T^ and *A. nanyangensis* HN36-1^T^) were analyzed using the OrthoFinder program, which classifies families of homologous proteins into orthogroups (Emms & Kelly, 2019). The genome information of all eight species selected in this study are presented in **S3**. A total of 13,079 proteins derived from a combined set of nine genomes were submitted to OrthoFinder, and 12,186 genes (93.2%) were assigned into 1,723 orthogroups, while the remaining 893 genes (6.8%) fell under the ‘unassigned genes’ category. As illustrated in UpsetR plot in Fig. 7A, a total of 755 orthogroups were assigned to the core orthogroups and 648 as accessory orthogroups. Subsequently, the distribution of orthogroups between strain Sy-1, *Acetilactobacillus jinshanensis* HSLZ-75^T^, and the seven strains species derived *Apilactobacillus* were explored. *Acetilactobacillus jinshanensis* HSZL-75^T^ has the highest number of species-specific orthogroup, 307 followed by strain Sy-1 which was 215. Noticeably, strain Sy-1 and species from the genus of *Apilactobacillus* shared a higher number of uniquely shared orthogroups (155) than the combined strain Sy-1 and *Acetilactobacillus jinshanensis* HSLZ-75^T^ (24 uniquely shared orthogroups). To better understand the strain Sy-1’s unique properties, all 215 unique orthogroups consisting of 264 genes were further classified using the EggNOG database. However, this resulted in most orthogroup (94 genes) being classified under ‘category S: function unknown’, requiring further exTable erimental work on functional gene validation is necessary. Otherwise, most orthogroups belonged to the amino acid transport metabolism (17 genes) category, followed by coenzyme transport and metabolism (15 genes) (Fig. 7B).

#### Carbohydrates metabolism

**Figure 8:**
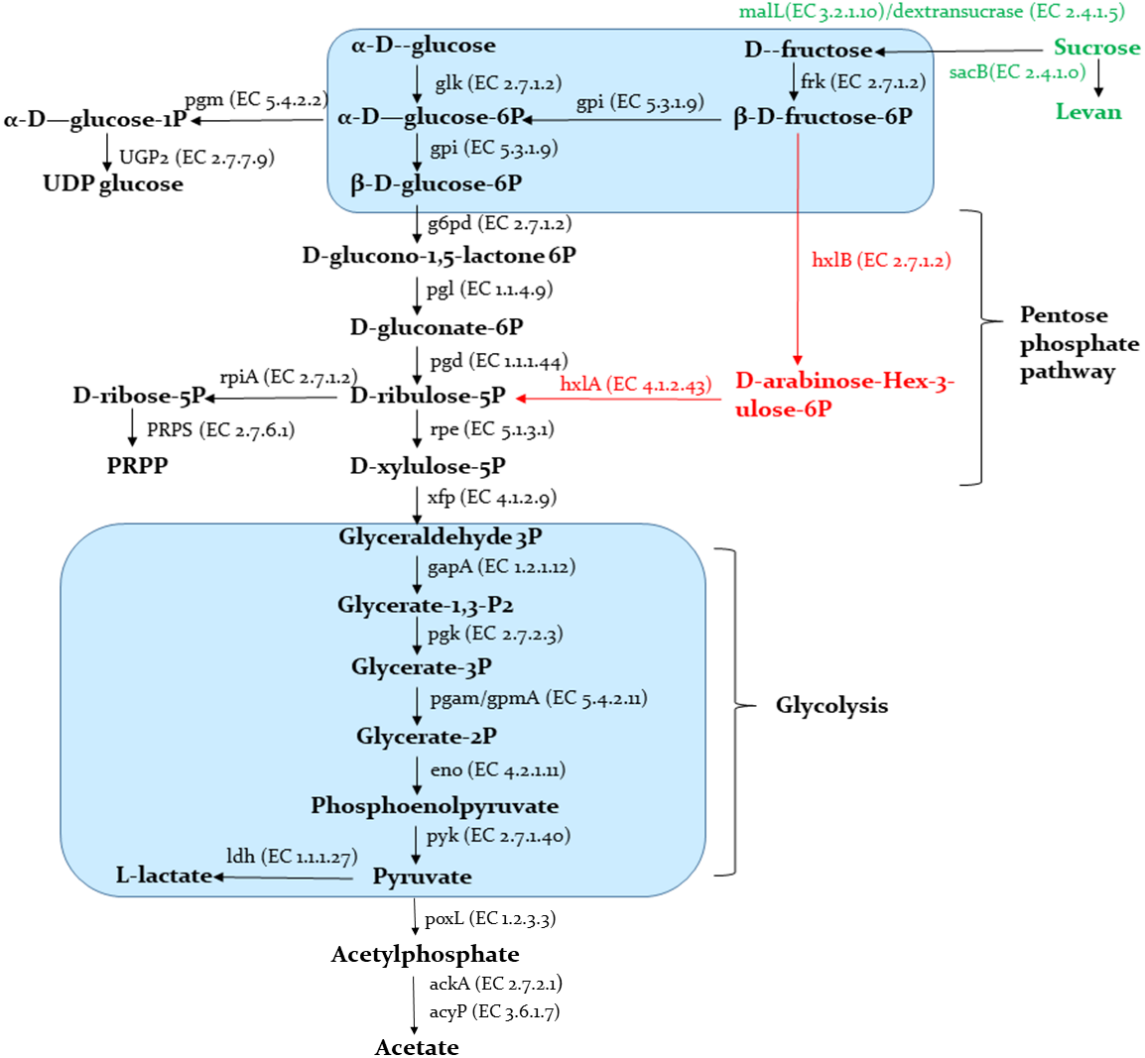
Schematic representation of carbohydrates metabolism. Pathways indicated in black are present in strain Sy-1, all species in the genera *Apilactobacillus* spp. and *Acetilactobacillus jisnshanensis*. Pathways indicated in red are only present in strain Sy-1, *Apilactobacillus timberlakei, Apilactobacillus quenuiae*, and *Apilactobacillus micheneri*.

A schematic representation of the carbohydrates metabolism predicted in the Sy-1 and other genomes are provided in Fig. 8. The results showed that Sy-1 is equipped with genes involved in major pathways of central carbon metabolism, including glycolysis and the pentose phosphate pathway. Genes encoding for phosphofructokinase and fructose-biphosphate aldolase were absent in the Sy-1 genome. These are the key enzymes for the Embden-Meyerhof pathway, and other carbohydrates metabolism are involved in the homofermentative metabolism of lactic acid. As a fructophilic bacterium, strain Sy-1 can synthesize ribose-5-phosphate from glucose *via* the pentose phosphate pathway. This yields phosphoribosyl pyrophosphate (PRPP), a precursor of purine pyrimidine and histidine metabolism. The author also noticed that strain Sy-1 could synthesize ribose-5-phosphate through D-arabino-hex-3-ulose-6-phosphate intermediate (M00580) due to the presence of rpiA, hxlA, and hxlB genes. This pathway also exists in the three species of *Apilactobacillus* viz *A. timberlakei, A. quenuiae,* and *A. micheneri.*

Consistent with Sy-1’s ability to use fructose, glucose, and sucrose as sole carbon sources, genes involved in the catabolism of these hexoses were present in the metabolic network of the bacterium. Pertinently, when compared to *Acetilactobacillus jinshanensi* s and *Apilactobacillus* spp., strain Sy-1 might have the unique ability to produce levan, based on the presence of sacB gene that encodes for the enzymes levansucrase. Conversely, pathways required for the catabolism of ribose, xylose, gluconate, and galactose were absent or incomplete. The data is consistent with the inability of strain Sy-1 to utilize these carbohydrates, as demonstrated by API50CHL analysis. Strain Sy-1 could synthesize uridine diphosphate glucose (UDP-glucose) and uridine diphosphate N-acetylglucosamine (UDP-Glc-Nac). Similar to other FLAB members, the Sy-1 genome did not encode an intact citrate acid cycle (TCA) (**S4**). However, two of the enzymes in this pathway, fumarate reductase, and fumarate hydratase are present. Sy-1 also has a complete isoprenoid biosynthesis pathway. The corresponding isoprenoids enzyme present in the genome of strain Sy-1 includes isopentenyl-diphosphate delta isomerase and geranylgeranyl diphosphate synthase.

Nectar sugars and polysaccharides present in the honey and pollen have to be broken down before further utilization as energy sources. CAZymes encoded by the honey LAB genome play a key role in breaking down carbohydrates into smaller components. The analysis CAZymes revealed the existence of 17 glycosyl hydrolases (GH), 16 glycosyl transferases (GT), and 1 polysaccharide lyases (PL) in Sy-1 (**S5**). The GH annotation in the Sy-1 genome includes GH13, and GH32 denote oligo-1,6-glucosidase and β-fructofuranosidase and fructan beta-(2,1)-fructosidase, and GH3 represents β-N-acetyl hexosaminidase . Interestingly, PL1 represents pectate lyase that digests polygalacturonic acid (PGA) backbone of pectin and debranching enzymes, were identified in the Sy-1 genome. Pectin constitutes a major component of plant cell walls and makes up the different pollen walls layers (Moran, 2015). Pectate lyase was also found in honey bee-associated bacteria such as *A. micheneri*, *A. quenuiae*, and *Gilliamela* spp. (Ludvigsen et al., 2018; Vuong & McFrederick, 2019).

Strain Sy-1 possessed only two PTS genes, phosphoenolpyruvate-protein phosphotransferase (ptsI) and phosphocarrier protein HPr (ptsH). Hence, this might account for their poor carbohydrate metabolism and solely rely on the MFS superfamily’s ABC-transporters and/or secondary transporters. This genetic characteristic is similar to FLAB members, *A. apinorum, A. kunkeei, A. micheneri, A. quenuinae, A. ozensis,* whose posseses either one or two PTS genes **(S4).**

#### Genome stability

Genome stability is significant in determining microorganisms destined for food applications. This is due to the possible horizontal transfer of pathogenic or antimicrobial resistance genes. Therefore, the presence of prophages, genomic islands (GIs), and insertion elements should be investigated. Sy-1 enclosed only one incomplete prophage (46.9 kb) that resembled PHAGE_Bacill_G_(NC_023719). The incomplete prophage region extended from 976957 bp to 1023917 bp and contained 31 CDS (**S6**). Genomic islands (GIs) are the segments of DNA that represent horizontal gene transfer in a bacterial genome. Natural competence and bacteriophage infection are two processes that might result in horizontal gene transfer between bacteria (Chen et al., 2019). One GI was identified in the genome of Sy-1. GI-1 represents seven genes, including transposase and six hypothetical proteins (**S7**). These are likely to be acquired genes, which is an essential factor in the “novelty” of this strain. We also identified double copies of insertion elements from the IS1182 family, and both elements were orthologues to ISP2 of *Lactobacillus plantarum*. Plasmids screened by PlasmidFinder fail to detect any plasmid sequence in the Sy-1 genome. Moreover, no plasmid replication protein (repA) was identified in the genome of Sy-1. CRISPRs are a family of DNA repeats that function as an adaptive immune system by defending against foreign genetic elements. Three CRISPR-associated sequences, Cas1, Cas2, and Cas9, were identified in the genome of Sy-1. Thus, the finding confirms the importance of resistance to exogenous DNA in this species.

#### Strain Sy-1 harbors folic acid and riboflavin production genes

Folates, a group of water-soluble vitamins, have become extremely important in the diet (Hanson & Gregory III, 2011). Folate deficiency has been linked to several serious illnesses, including chronic heart failure and neural tube defects (Czeizel et al., 2013). Several studies have shown that certain LAB strains can synthesize natural folate (Rossi, Amaretti & Raimondi, 2011), indicating their importance as sources of folic acid. Folate biosynthesis contains two metabolic branches; the first is to convert guanosine triphosphate (GTP) to 6-hydromethyl-7,8-dihydropterin, while the second branch converts chorismite to para-aminobenzoic acid (pABA) (Magnúsdóttir et al., 2015). In the Sy-1 genome, genes involved in pABA biosynthesis and the GTP pathway (**S8**) were present. However, Sy-1 lacked the folQ genes encoding 7,8-dihydroneopterin aldolase, the second enzyme for converting GTP to tetrahydrofolate (THF). Folate incomplete biosynthesis pathway was also revealed by KEGG tools, which pointed again to the same missing enzymatic block in the Sy-1 genome. To confirm these results, the gene folQ coding for probable dihydropterin triphosphate pyrophosphorylase from the genome of *Lactococcus lactis* subsp*. cremoris* (strain NZ9000) was used as a reference and blasted against the genome of Sy-1. The study found 50.82% similarity with the genes annotated as NUDIX domain-containing protein (HZL42_05080). This gene has no KO assigned, which explains the failure of BlastKOALA to detect the folQ gene. The HPLC chromatogram verified folate biosynthesis by strain Sy-1 at 1.346 mg/L, and the value is higher than those reported for *L. plantarum* strains isolated from sourdough (16–143 ng/mL) and maize (30 to 55 ng/mL) (Carrizo et al., 2016; Rodrigo-Torres et al., 2019). Therefore, the ability of Sy-1 to produce folates, implying the bacterium’s biotechnological importance for the development of novel products naturally enriched in folates.

Unlike certain plants, fungi, and bacteria, humans cannot synthesize riboflavin and must obtain it by dietary supplementation (Burgess, Smid & Van Sinderen, 2009). Riboflavin is a key component of cellular metabolism since it is the counterpart to the coenzymes flavin mononucleotide (FMN) and flavin adenine dinucleotide (FAD, where both are hydrogen carriers in a variety of biological redox reactions (Wolf, 2008). This study discovered a complete riboflavin operon, ribBEHF, in the Sy-1 genome. The KEGG tools also subscribed that riboflavin biosynthesis from GTP is complete (**S9**). The respective HPLC analysis further confirmed that strain Sy-1 produces riboflavin at 0.035 mg/L of riboflavin after 24 h of incubations in a culture medium. The values are lower than the reported riboflavin overproducer strain *L. fermentum* PBCC11.5 (1.3 mg/L). After 48 h, riboflavin was no longer detectable. The outcome seen here correlates with Sy-1 harboring the ribC that converts riboflavin to two active forms, namely flavin mononucleotide and flavin adenine nucleotide.

#### Antibiotics and metal resistance

As antibiotic resistance genes raise concerns in both health and biotechnology applications, Sy-1 was examined for antibiotic resistance genes. After blasting against Resfam, ResFinder, ARDB, and CARD databases, no antibiotic resistance genes were found. The antibiograms revealed that Sy-1 was sensitive against ciprofloxacin, tetracycline, chloramphenicol, and gentamicin but resistant to sulphafurazole. Sulphafurazole is a short-acting sulfanilamide and a synthetic analog of para-aminobenzoic acid (PABA) with broad-spectrum anti-bacterial properties.Sulphafurazole is a folate antagonist, in which they compete with PABA for the enzyme dihydropteroate synthase, thereby preventing the incorporation of PABA into dihydrofolic acid. The high production of folate (folic acid) by Sy-1 might function as a resistance mechanism against sulphafrazole. High folate pools are expected to compete more efficiently with sulphafurazole for binding to dihydropteroate synthase. KEGG tools also revealed the anti-folate resistant pathway.

Sy-1 strain appears to be adept at detoxification, as signatures of toxin tolerance were found in the genome. Toxins such as heavy metals and biphenyl can accumulate in flowers and, therefore likely to occur in pollen provisions and honey (Vuong & McFrederick, 2019). Genome mining showed that strain Sy-1 is equipped with genes resistance to heavy metals such as copper, cadmium, and zinc, along with the genes responsible for biphenyl degradation protein (**S10**). Strain Sy-1 also possesses a multidrug-efflux transporter and metal-extruding pumps that could extrude metal toxins and antibiotics to enable flexibility and growth in the presence of noxious compounds. Finally, Sy-1 may be competitive under antibiotic or nutrient-limiting stress due to five toxin-antitoxin genes present in the genome.

### Conclusions

This study successfully outlines the genetic background of strain Sy-1 isolated from freshly collected Malaysian *H. itama* honey. The taxonomic analyses placed strain Sy-1 as a novel genus in the family of *Lactobacillacea*. The preference of strain Sy-1 to D-fructose over D-glucose and partial deletion of *adhE* gene further confirmed that Sy-1 is an obligate FLAB bacterium. Genome annotation of Sy-1 divulged genes related to carbohydrate transport and metabolism, prophages and CRISPR adaptive immunity, multidrug and metal resistance, and folic acid and riboflavin biosynthesis. The genome information coupled with experimental studies supported the ability of strain Sy-1 to produce high folic acid. Comparative analysis between strain Sy-1 and the *Apilactobacillus* and *Acetilactobacillus jinshanensis* species shows the former carries 264 unique genes, mostly related to the amino acid metabolism and coenzyme transport and metabolism. Thus, our findings provide the genetic basis of strain Sy-1 and its consideration as a potential novel health-promising organism. However, it is necessary to further unravel the specific health benefits of the Sy-1 bacterium through *in-vivo* experiments.

## Supplemental Information

10.7717/peerj.13053/supp-1 Supplemental Information 1 The functional annotation of genome Sy-1 predicted using the PGAP annotator, Egg-nog mapper, and KEGG database

10.7717/peerj.13053/supp-2 Supplemental Information 2 The complete genome sequence of strain Sy-1

## Additional Information and Declarations

### DNA Deposition

The following information was supplied regarding the deposition of DNA sequences: The 16S rRNA gene and the whole-genome sequence of strain Sy-1 are available at NCBI: MW715007 and JACCIU000000000, respectively. The culture of Sy-1 was deposited in the Microbial Culture Collection (UNICC), Institute of Bioscience, Universiti Putra Malaysia: UPMC 1412.

### Data Availability

The following information was supplied regarding data availability: The functional annotation of genome Sy-1 predicted using the PGAP annotator, Egg-nog mapper, and KEGG database are available in the Supplemental File.
